# Influence of Cellular Trafficking Pathway on Bluetongue Virus Infection in Ovine Cells

**DOI:** 10.3390/v7052378

**Published:** 2015-05-13

**Authors:** Bishnupriya Bhattacharya, Cristina C. Celma, Polly Roy

**Affiliations:** Department of Pathogen Molecular Biology, Faculty of Infectious and Tropical Diseases, London School of Hygiene and Tropical Medicine, Keppel Street, London WC1E 7HT, UK; E-Mails: priya.bhattacharya@lshtm.ac.uk (B.B.); cristina.celma@lshtm.ac.uk (C.C.C.)

**Keywords:** late domain, multivesicular body, ubiquitin, BTV, trafficking, release, NS3

## Abstract

Bluetongue virus (BTV), a non-enveloped arbovirus, causes hemorrhagic disease in ruminants. However, the influence of natural host cell proteins on BTV replication process is not defined. In addition to cell lysis, BTV also exits non-ovine cultured cells by non-lytic pathways mediated by nonstructural protein NS3 that interacts with virus capsid and cellular proteins belonging to calpactin and ESCRT family. The PPXY late domain motif known to recruit NEDD4 family of HECT ubiquitin E3 ligases is also highly conserved in NS3. In this study using a mixture of molecular, biochemical and microscopic techniques we have analyzed the importance of ovine cellular proteins and vesicles in BTV infection. Electron microscopic analysis of BTV infected ovine cells demonstrated close association of mature particles with intracellular vesicles. Inhibition of Multi Vesicular Body (MVB) resident lipid phosphatidylinositol-3-phosphate resulted in decreased total virus titre suggesting that the vesicles might be MVBs. Proteasome mediated inhibition of ubiquitin or modification of virus lacking the PPXY in NS3 reduced virus growth. Thus, our study demonstrated that cellular components comprising of MVB and exocytic pathways proteins are involved in BTV replication in ovine cells.

## 1. Introduction

Cell membranes present significant dissemination barriers and viruses have developed sophisticated mechanisms for entering and exiting cells. While enveloped viruses bud through membranes, non-enveloped viruses are believed to be predominantly released by cell lysis. However, recently alternate routes involving non-lytic pathways of exit have been noted for non-enveloped viruses [1,2,3,4].

Bluetongue virus (BTV), a member of the *Reoviridae* family, is a double capsid complex non-enveloped orbivirus that infects ruminants via biting gnats (*Culicoides* sp). Like other members of the family, BTV egress in mammalian cells predominantly occurs by cell lysis. However, BTV also exhibits budding morphology similar to enveloped viruses and can be released as enveloped particles [5,6].

Enveloped virus budding at cellular membranes is facilitated through viral recruitment of members of the cellular endosomal sorting complex required for transport (ESCRT) pathway that act in a sequential manner to form the multivesicular bodies (MVBs) [7]. The matrix proteins of a number of enveloped viruses possess highly conserved domains known as late domains that aid in binding to different components of ESCRT pathway and mediates virus release through budding [8]. While PSAP/PTAP domain recruits a component of the ESCRT-I complex (Tsg101) [8,9], the YPXL and LXXLF motifs bind to AIP-1/Alix and facilitate in bridging ESCRT-I and ESCRT-III complexes [8,10,11]. The third motif PPXY (where X can be any amino acid but is most commonly a proline residue) recruits host ubiquitin ligases by binding the WW domains present in members of the NEDD4 (Neural precursor cell Expressed Developmentally Down-regulated protein 4) family of HECT (homologous to the E6AP carboxyl terminus) ubiquitin E3 ligases [8,12]. Along with the ESCRT pathway, cellular ubiquitin has also been implicated as a sorting signal in transport of proteins from the Golgi to the endosomes [13] and for entry into the vesicles of MVB [14,15,16]. Ubiquitin also mediates release of enveloped viruses that encode PT/SAP or PPXY late domains [17,18,19]. In addition to ubiquitin, phosphoinositide, the phosphorylated derivatives of phosphatidylinositol (PtdIns), a membrane phospholipid are also known to be involved in the MVB pathway, particularly in the biogenesis of MVB vesicles [20,21,22,23]. PtdIns is distinctive among phospholipids in that its inositol head group features hydroxyl groups that can be modified reversibly by phosphorylation at the 3', 4' or 5' position, either singly or in combination, by a family of PtdIns kinases that are localized in the cytoplasm. Among the seven unique phosphoinositides that are known to be synthesized in cells, PtdIns(3)P formed by phosphatidylinositol 3-kinase (PI3K) is enriched in endosomal membranes and on the luminal vesicles of MVBs [24].

The four major BTV structural proteins are arranged in two capsids; the outer capsid composed of VP2 and VP5 encloses an inner capsid or core composed of two other major proteins VP3 and VP7 [25]. The core contains enzymatic proteins that are closely associated with the 10 genomic double-stranded RNA (dsRNA) segments. In infected cells BTV synthesizes four non-structural proteins (NS1-NS4), one of which (NS3) is glycosylated and is associated with intracellular membranes and plasma membranes [26,27,28,29]. Using alternate initiator methionine residue, NS3 is expressed as a full-length protein and as a truncated variant (NS3A) that lacks the initial 13 residues from the amino terminal end of the protein [29]. A recent study showed that a mutant virus expressing only NS3 but not NS3A, was capable of efficient growth and release from mammalian cells [30]. In contrast, BTV expressing only NS3A was severely attenuated [30]. NS3 is a relatively small protein (228 amino acids), with a putative long N-terminal domain and a shorter *C*-terminal cytoplasmic domain that are connected by two transmembrane domains and a short extracellular domain [31]. While the cytoplasmic amino terminal end of NS3 interacts with cellular component p11 (S100A10), a subunit of the calpactin complex of exocytic pathway, the cytoplasmic carboxy terminal end of NS3 interacts with VP2, the most exposed viral capsid protein [30,32]. Perturbation of NS3-p11 or NS3-VP2 interaction has been reported to drastically alter virus trafficking and release [6]. In addition, despite being a non-structural protein, NS3 also possesses PTAP and PPXY domains, of which PTAP has been shown to influence BTV release in BSR (derived from BHK-21) cells, that are used routinely for BTV growth in lab [6,33]. However, whether ubiquitin and late domains, in particular PPXY, have a functional role in BTV trafficking or release in natural host cell context has not been investigated to date.

In this study we have undertaken a comprehensive study on involvement, if any, of ubiquitin and PPXY late domain in BTV trafficking and release, particularly in cells derived from natural host sheep (PT cells derived from sheep kidney). Electron microscopy of infected PT cell sections showed a striking vesicular distribution of the virus particles, indicating that vesicular structures are involved in virus replication. Further, recovery of modified virus particles containing mutations in PPXY motif also influenced virus release, accompanied by altered localization of NS3 and virions. Thus, ubiquitin and PPRY domain in BTV influences virus growth in cultured sheep cells, derived from the natural host of BTV. This indicated that the interplay of vesicles, ubiquitin and NEDD4 family of proteins are involved in BTV maturation, trafficking and egress similar to that of enveloped viruses.

## 2. Materials and Methods

### 2.1. Cell Lines, Virus and Antibodies

BSR (derivative of baby hamster kidney cell), BSR/NS3 and PT (ovine kidney) cell lines were cultured as described [6,34]. Wild type BTV1 (South African Strain), BTV1Δ_PTAP_ [6] and BTV1Δ_PPRY_ were propagated and titred in BSR cells.

All antibodies against BTV proteins NS3, NS2 and VP5 were generated in our laboratory [35,36]. Actin and ubiquitin were immunolabeled with mouse monoclonals (Sigma Aldrich and AbCam, Cambridge, UK). Fluorescently labelled secondary antibodies (Alexa Fluor 488, Alexa Fluor 546) and Hoechst were obtained from Life technologies.

### 2.2. Plasmids and Site-Directed Mutagenesis

Site-directed mutagenesis was performed to mutate PPRY motif to AARA in BTV1 S10 sequence [37]. Briefly, two complementary primers were used for mutating the concerned residues in pUCBTV1T7S10 template; NS3_F, 5'-cCGTGTGGATGACACGATTTCCCAAGCGGCCCGGGCGGCTCCGAGTGCGCCTATGCCATCGTCGATGCC-3': NS3_R, 5'-GGCATCGACGGCCATAGGCGCACTCGGAGCCGCCCGGGCCGCTTGGGAAATCGTGTCATCCACACGG-3'. The mutated versions of NS3 were sequenced (MWG Biotech) to confirm the presence of the designed mutations.2.3 Recovery of tagged viruses.

The T7 BTV capped (BTV1S1-S9) and uncapped transcripts (BTV1S10, BTV1S10 with AARA mutation) were generated as described [38]. The mutant BTV particles were recovered following the method described previously [39]. Genomic dsRNA from cells infected with control or mutant BTV was analyzed as described [38] and cDNA copies of S10 from control or mutant BTVs were obtained by reverse transcription-PCR (RT-PCR) as describe before [38].

### 2.3. Virus Growth Kinetics and Virus Release

Monolayers of PT, BSR or BSR-NS3 cells were synchronously infected with WT BTV, BTVΔ_PPRY_ or BTVΔ_PTAP_ at multiplicity of infection MOIs of 0.1, 1 and 10. At 0, 12 and 24 h (hours) post infection (pi), the total titres were determined by plaque assay [40]. Virus release studies were undertaken by synchronously infecting monolayers of PT or BSR cells with WT BTV or BTVΔ_PPRY_ (both at 0.1 MOI). After absorption for 1 h, cells were washed with DMEM and incubated in growth media supplemented with 2% FCS for 12 and 24 h. The supernatants were collected and the cells were washed twice with fresh medium prior to harvest. The titre of each fraction was determined by plaque assay. While the supernatant represented released virus, the total virus titre was calculated as the sum of released and cell associated fractions [6]. Western blot was undertaken to monitor the expression of BTV proteins NS2 and NS3, while cellular protein actin was used as loading control. Each blotting experiment and plaque assay was repeated three times and the amount of protein expression was quantitated by ImageJ. The mean and standard error of the virus titres and intensities of the protein bands were calculated (Sigma Plot 2000; Systat Software Inc., San Jose, CA, USA) and the *p* values were also determined by paired t test in Excel (Microsoft).

### 2.4. BTV Release Assay

PT and BSR cells seeded in six-well plates were infected with BTVΔ_PPRY_ or BTV at 0.1 MOI. At 12 or 16 h pi the supernatants were harvested and processed for virus release as described [33]. To examine the intracellular expression of BTV proteins, whole-cell lysates were analyzed in parallel by western blot. The cell release fractions concentrated as described were infected in BSR cells and expression of BTV protein was analyzed by western blot.

### 2.5. Ubiquitin and PI 3-Kinase Inhibition Assay

Proteasome inhibitors MG132, Lactacystin and PI 3-Kinase inhibitor LY294002 were obtained from Sigma Aldrich. For PI 3-Kinase inhibition, synchronously infected (1 MOI) PT and BSR cells were incubated for 12 h and then supplemented with 100 µM of LY294002 for six more hours of incubation. In ubiquitin inhibition assay synchronously infected PT and BSR cells (0.1 MOI) were either incubated for 12 and 16 h in growth media plus 10 µM of inhibitors or initially incubated for 10 and 14 h without any inhibitor and then supplemented with 20 µM of inhibitors for two more hours of incubation. Untreated infected cells and cells treated with DMSO, the diluent for the inhibitors were used as controls. The treated and untreated cells were either processed for plaque assay, protein production or confocal microscopy. In parallel, control uninfected cells were also treated similarly with the respective inhibitors in cell viability assays to ascertain the percentage of live cells.

### 2.6. Confocal Microscopy

PT and BSR cells synchronously infected with BTVΔ_PPRY_ or control BTV at 0.1 MOI were processed for confocal microscopy on a Zeiss LSM 510 at 12 and 24 h pi as described [40]. The images were obtained using LSM 510 image browser software and processed using Photoshop Element 8.0 software (Microsoft). Each set of fixed cell experiments were repeated at least three times and fields containing 60 cells in average were statistically analyzed for distribution of both wild type and mutated NS3. The mean and standard error of percentage of difference in NS3 localization were calculated (Sigma Plot 2000; Systat Software Inc.) and the *p* values were also determined by Excel (Microsoft).

### 2.7. Electron Microscopy

PT and BSR cells infected at 0.1 or 1MOI were processed for cell sectioning at 24 or 48 h pi as described [6].

## 3. Results

### 3.1. Vesicular Distribution of BTV Particles in Sheep Cells

**Figure 1:**
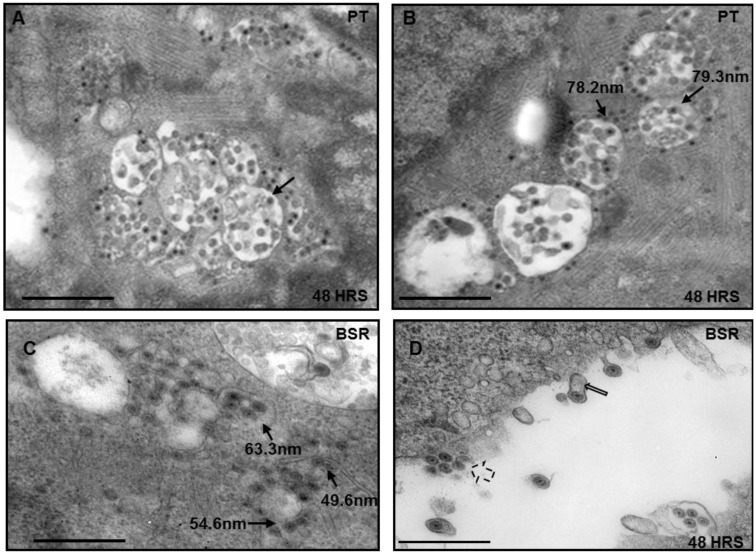
Electron Microscope analysis of infected cell sections. Both PT and control BSR cells were infected with Bluetongue virus (BTV), fixed and processed for cell sectioning. Virus particles in PT (**A**,**B**) and BSR (**C**,**D**) cells are associated with vesicles (thin black arrows) or released and attached to membranes (open black arrows). In BSR cells the particles can also be seen released by the perturbation of the plasma membrane (broken arrows). Bar, 500 nm.

Intracellular distribution of BTV was analyzed to understand the growth phenotype of the virus in PT cells that have been derived from sheep kidney, the natural host of BTV. For this purpose, initially electron microscopy of ultrathin sections of PT cells infected with BTV (serotype 1) at MOI of 1 were analyzed for cytoplasmic distribution of BTV particles at 48 h pi (Figure 1). For comparison, BSR cells that are routinely used for BTV growth in the laboratory were used in parallel. In PT cells, a clear distribution of negatively stained virus particles measuring around 70 nm in diameter was observed within intracellular vesicles (Figure 1A,B, thin black arrows). Infected BSR cells also showed vesicular distribution of BTV at 48 h pi, but the particles within the vesicles were smaller (50 to 60 nm) and were much closer in size to BTV cores than mature virus particles (Figure 1C). In addition, majority of the particles were observed in the vicinity of membrane like structures in the cytoplasm and surrounding the vesicles (Figure 1C). Moreover, there were budding virus particles and the presence of membrane-like structures surrounding the released virus particles were visible (Figure 1D, thin open black arrows). Interestingly, the budding phenomenon was not observed in infected PT cells (results not shown). Cell sections of infected BSR cells also showed BTV release by perturbation of cellular membranes (Figure 1D, broken black arrows). These results are consistent with previous reports of BTV budding from BSR cells and the presence of membranes that surround released particles [6,28].

The vesicular distribution of BTV particles in sheep cells and subsequent localization of these vesicles in close proximity to the plasma membrane alluded to their importance in maturation and egress. Therefore, it was necessary to undertake further experiments in order to ascertain the influence of the vesicles in BTV replication.

### 3.2. Disruption of MVB and BTV Growth

MVBs have been reported to play an essential role in the assembly of enveloped viruses [41,42]. Since, inhibition of PI3K activity blocks MVB-vesicle formation [20,21], LY294002, a PI 3-Kinase inhibitor was used to investigate the effect of MVB disruption on BTV growth. Cell viability test with 30–100 µM of LY294002 demonstrated that up to 90% of PT and BSR cells could tolerate LY294002 at 100 µM (results not shown).

**Figure 2:**
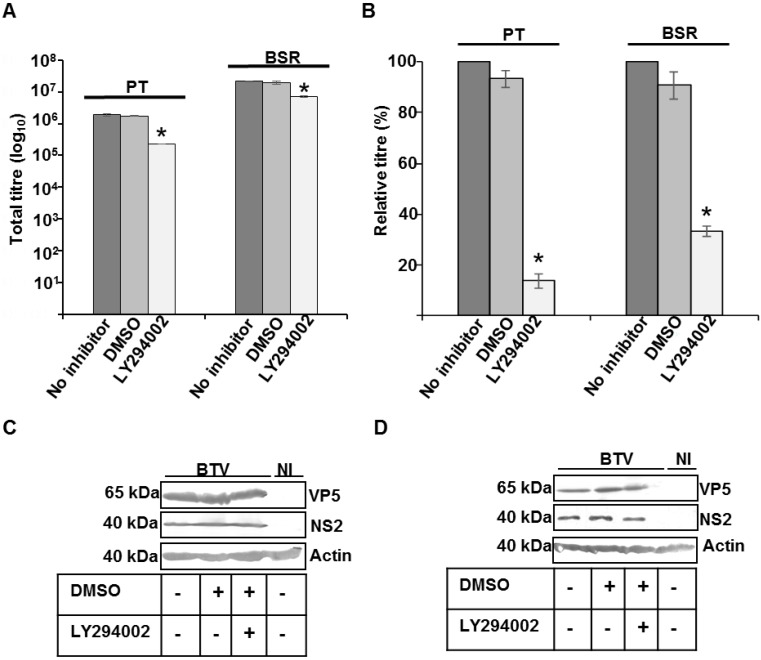
Effect of MVB disruption on BTV. (**A**,**B**) PT and BSR cells infected with WT BTV at 0.1 MOI were incubated at 12 h pi with LY294002, a specific inhibitor of PI 3-kinase. The total virus (**A**) and relative (**B**) titres at 16 h pi were plotted. The plaque assays were done in triplicate and bars represent standard error. Significant difference in the virus titres were designated by the asterisk (*); (**C**,**D**) Expression of NS2, VP5 and actin in infected PT (**C**) and BSR (**D**) cells. The cells were treated similar to A. Lysates were analyzed by SDS-PAGE and Western blotting. Molecular masses and proteins are indicated on left and right, respectively. Presence or absence of diluent DMSO and inhibitors has been indicated by (+) or (−). NI signified uninfected cells.

The effect of PI3K inhibition on total virus titre and protein production was assessed by infecting PT and BSR cells with WT BTV at 1 MOI for 12 h. Subsequently, the cells were washed and then further incubated for 4 additional hours in presence of 100 μM of LY294002. BTV is known to enter cells thorough endosomes [36,43] and it has been reported that the first cycle of BTV replication is completed within 12–16 h of infection [39,44]. Hence, in order to avoid the possible effect of LY294002 mediated inhibition of PI3K activity on virus entry and to restrict our study to the role of PI3K on BTV assembly and/or release, the infected cells were treated with the inhibitor between 12–16 h pi. Controls consisted of WT BTV infected cells incubated in the absence of LY294002 or incubated with DMSO, the diluent used for LY294002. Compared to the controls, LY294002 demonstrated a significant reduction in the total titres and the effect was more pronounced in PT cells (*p* < 0.01) than for BSR (*p* < 0.05) (Figure 2A). When the total titre was plotted as a relative percentage of the control virus titre that was not treated with DMSO or LY294002, a more significant reduction in the relative titre was also observed in repeated experiments for PT (*p* < 0.001) than BSR cells (*p* < 0.01) (Figure 2B). On average, infected and LY294002 treated PT cells exhibited a five times reduction relative to the control infected and non-treated cells, and the reduction was about 2.5 times for BSR (Figure 2B). An additional control comprising of infected cells treated with diluent DMSO showed no difference in relative virus titre to that of cells infected but not treated. However, the effect of PI3K inhibition on viral protein synthesis in Western blot (WB) showed equivalent expression of BTV VP5, the major outer capsid protein and NS2, a major non-structural protein, which is responsible for the formation of virus inclusion bodies (VIBs) in infected cells treated with LY294002, untreated cells and cells treated with the diluent (Figure 2C,D). Cellular actin was used as the loading control. Since, PI3K inhibition did not perturb viral protein synthesis but there was a significant decrease in the virus titre, these data indicated that the integrity of MVBs might be important for BTV growth. In addition, the integrity of the MVBs appeared to be more important in sheep cells (PT) than BSR cells.

### 3.3. Ubiquitin, the Cellular Partner for Trafficking of BTV to Vesicles

A wealth of data supports the view that ubiquitin serves as sorting signal for targeting of membrane proteins into the degradative MVB/lysosomal pathway [45]. Since BTV appears to be closely associated with cellular membranes and vesicular structures that might be MVB, the role of ubiquitin pathway was also investigated for its involvement in BTV replication and trafficking. It has been well established that inhibition of proteasomes results in decrease in the levels of cellular free ubiquitin [46]. Hence, two proteasome inhibitors, one which is a highly specific inhibitor of proteasome, such as lactacystin and the other, MG132, a tripeptide aldehyde which is less specific and can also inhibit pathways other than proteasomes, were used to explore the effect of ubiquitin inhibition on BTV replication and release [47,48]. Initially, a dose dependent experiment with different concentrations of MG132 and lactacystin was undertaken to evaluate the cell viability of treated cells. The results showed that the cell survival was greater than 90% after treatment with MG132 or lactacystin at concentrations ranging between 10–20 μM (results not shown).

**Figure 3:**
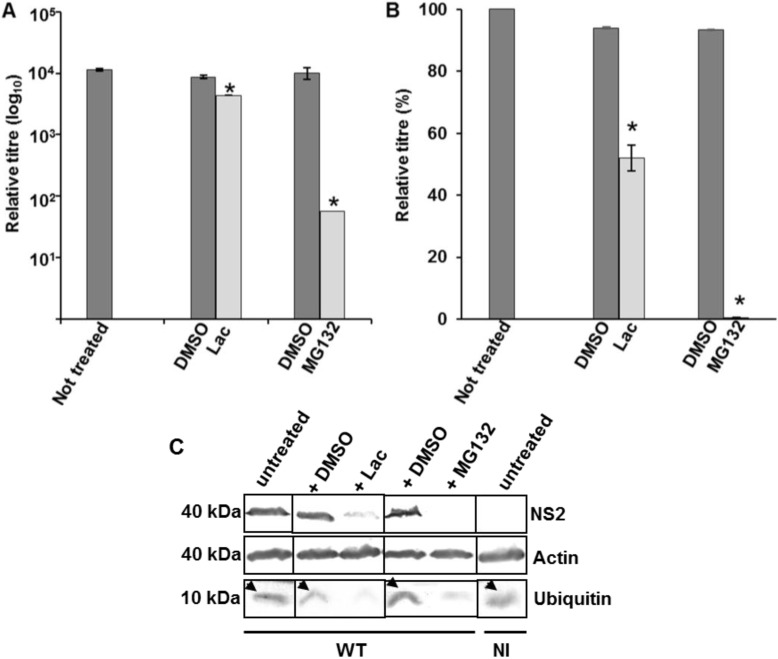
Influence of proteosome inhibitors on BTV growth in PT cells. (**A**,**B**) Cells infected with WT BTV at 0.1 MOI were incubated with MG132 or lactacystin. The total (**A**) and relative (**B**) titres were assessed at 12 h pi. The plaque assays were done in triplicate and bars represent standard error. Significant difference in the virus titres were designated by the asterisk (*). Presence or absence of diluent DMSO and inhibitors have been indicated below the graph; (**C**) Expression of NS2, actin and ubiquitin in treated and infected PT cells. The cells were treated similar to A. Lysates were analyzed by SDS-PAGE and Western blotting. Molecular masses and proteins are indicated on left and right, respectively. Arrows represent presence of ubiquitin in untreated cells. Presence or absence of diluent DMSO and inhibitors has been indicated on the top of the panel. NI signified uninfected cells.

The effect of proteasome inhibitors on total virus titre and protein production was assessed by infecting PT cells with WT BTV at 0.1 MOI in the presence of MG132 (10 µM) or lactacystin (10 µM) for 12 or 16 h. Controls consisted of WT BTV infected cells incubated in the absence of any inhibitor or incubated with DMSO, the diluent used for the inhibitors. Compared to the controls, both MG132 and lactacystin demonstrated a significant reduction in virus titres and the effect was more pronounced for MG132 (*p* < 0.01) than for lactacystin (*p* < 0.05), the highly specific inhibitor (Figure 3A,B). Both inhibitors also affected BTV protein synthesis as demonstrated by a significant decrease (*p* < 0.05) in the expression of BTV NS2 (Figure 3B). Similar to virus growth titres, the effect was more pronounced for MG132 (no NS2 detected) than for lactacystin (<81.7% ± 2.9 reduction) (Figure 3C). Since in the presence of inhibitors, there was also a significant decrease (81% ± 0.03) in free ubiquitin (*p* < 0.05), the reduction in virus titre and protein production could therefore be attributed to the lack of free ubiquitin. Subsequently, the total infected cell lysate obtained in the presence or absence of inhibitors was analysed for ubiquitination pattern in WB by ubiquitin antibody. However, no significant difference was observed in the ubiquitination pattern of infected cells that were treated or not treated with the inhibitors (results not shown). As VIBs act as factories for BTV core production, the first step in mature virus particle formation [49], a significant reduction in production of NS2 and corresponding decrease in virus titre indicated that the effect of ubiquitin might have an essential role in the formation of inclusion bodies. Alternatively it is possible that an active ubiquitin-proteasome machinery, similar to Rotaviruses, is necessary for BTV replication [50].

**Figure 4:**
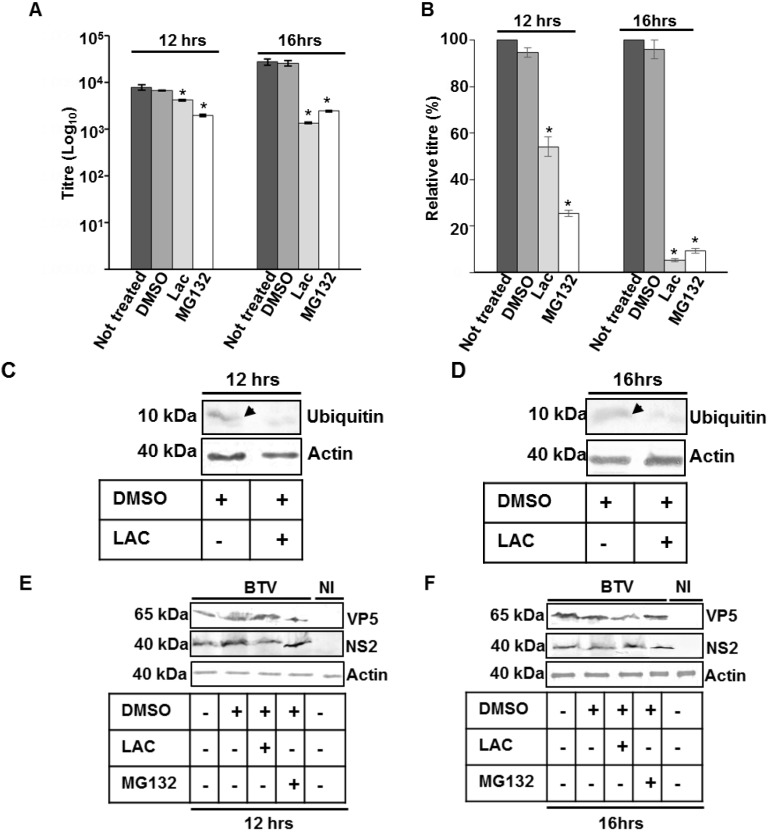
Influence of proteosome inhibitors on virus release. (**A**) PT cells infected with WT BTV at 0.1 MOI were incubated for 10 or 14 h and treated with MG132 or lactacystin for 2 h. Total and released virus titres were assessed by plaque assay and the significant difference in the virus titres were designated by asterisk (*). The plaque assays were done in triplicate and bars represent standard error; (**B**) Relative virus titres of (**A**); (**C**–**F**) Expression of free ubiquitin (**C**,**D**) and VP5, NS2, actin (**E**,**F**) in treated and infected PT cells. The cells were treated similar to **A**,**B**. Lysates were analyzed by SDS-PAGE and Western blotting. Molecular masses and proteins are indicated on left and right, respectively. Presence or absence of diluent DMSO and inhibitors has been indicated by (+) or (−), respectively. Arrows represent presence of ubiquitin in untreated cells. NI signified uninfected cells.

Subsequently, another set of experiments were designed to specifically probe the effect of ubiquitin inhibition on the later stages of BTV maturation and or release. For this purpose, PT cells infected with BTV at low MOI, were incubated for only 10 or 14 h and then further incubated for 2 additional hours in presence of 20 μM of MG132 or lactacystin (Figure 4). Analysis of total virus titres in presence of inhibitors demonstrated that compared to cells that were either untreated or treated with DMSO, MG132 and lactacystin significantly decreased the total viral titres at 12 and 16 h pi (Figure 4A). While at 12 h pi the effect was more pronounced for MG132 (*p* < 0.01) than lactacystin, (*p* < 0.05), at 16 h pi the decrease for the two inhibitors (*p* < 0.05) was equivalent. In addition, when the inhibitor induced decrease in virus titre was plotted as a relative percentage to that of infected cells that were not treated with inhibitors (Figure 4B), MG132 (*p* < 0.001) again demonstrated a more significant decrease than lactacystine (*p* < 0.01) at 12 h pi. However, at 16 h pi the relative decrease was similar for both the inhibitors. Monitoring MG132 and lactacystin mediated inhibition of free cellular ubiquitin demonstrated significantly lower (*p* < 0.05) level of ubiquitin (<70% ± 0.03) than untreated cells or cells incubated with DMSO (Figure 4C,D). As before, no significant difference was observed in the ubiquitination pattern of infected cells that were treated or not treated with the inhibitors (results not shown). Measuring the virus protein production also did not show any significant difference (*p* > 0.05) in NS2 or VP5 synthesis at 12 (Figure 4E) and 16 (Figure 4F) h pi in cells that were treated with lactacystin or MG132 (Figure 4E,F) and cells that were either untreated or treated with DMSO. There was also no difference in the expression of actin, the loading control (Figure 4C–F). Since in this assay ubiquitin inhibition had no effect on virus protein production but still affected total titre at early time points (12 or 16 h pi), this indicated that influence of proteasome inhibitors on BTV replication might be either linked to the maturation of complete virus particles and or to an early non-lytic release of BTV particles.

### 3.4. Influence of PPRY Late Domain on BTV Distribution

Two completely conserved late domain motifs (PSAP and PPRY) have been detected in NS3 of all BTV serotypes [33]. The abrogation of BTV release by the disruption of NS3 PSAP motif has already been studied [6]. Since, the second late domain motif PPRY is known to act as ubiquitinylation substrates of NEDD4 family members that are sorted through the MVB pathway [51], site specific mutations in replicating viral genome were undertaken to analyze the importance of this domain in BTV distribution and release.

**Figure 5:**
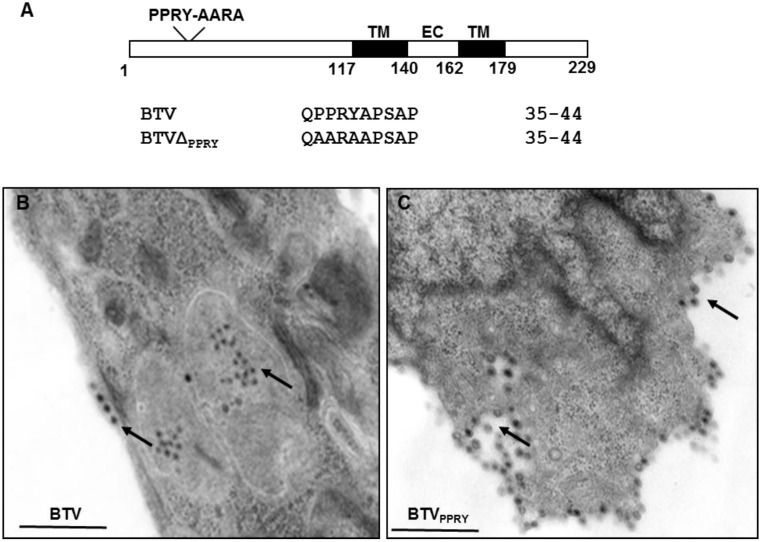
Mutation of PPXY late domain in NS3. (**A**) Schematic representation depicting position of the PPRY motif in NS3. The numbers designate amino acid positions in NS3 sequence; TM and EC signifies transmembrane domain and extra cellular regions (**B**,**C**). Electron Microscope analysis of PT cell sections infected with WT BTV (**B**) and BTVΔ_PPRY_ (**C**) analyzed at 20 h pi. Virus particles under the plasma membrane and within vesicles (black arrows) been indicated. Bar, 500 nm.

Structural studies have shown that the first two proline and the last tyrosine residues in the PPXY late domain are important for binding with the WW domains present in members of the NEDD4 family of HECT ubiquitin E3 ligases [12]. Since all three residues (P_37_P_38_Y_40_) are completely conserved in the NS3 sequences of 27 BTV serotypes, site-directed mutagenesis was undertaken to substitute both proline and tyrosine residues by alanine (PPRY-AARA) (Figure 5A). As described previously [39], uncapped S10 T7 transcripts (BTV1-S10) were generated for the mutant AARA domain and BTV RG system was used to recover the mutant BTV1Δ_PPRY_ virus in BSR cells. Analysis of the genomic dsRNA pattern did not show any detectable difference between WT BTV and the recovered BTV1Δ_PPRY_ virus (not shown). Sequencing of BTV1Δ_PPRY_ virus also confirmed the presence of PPRY to AARA mutation at the relevant position in the S10 segment of the mutant BTV1Δ_PPRY_ virus (results not shown).

To determine whether the PPRY domain in NS3 plays an active role in virus trafficking in sheep cells, PT cells infected with 0.1 MOI of BTVΔ_PPRY_ or WT BTV were processed for cell sectioning at 20 h pi. PT cells infected with WT BTV demonstrated particles present within vesicles and also underlying the cell membrane (arrow) (Figure 5B). Unlike WT BTV infected cells, majority of BTVΔ_PPRY_ mutant virus particles (black arrow) were not seen within vesicles, rather outside the vesicles and surrounding them (Figure 5C). In addition, compared to WT BTV, a more concentrated accumulation of particles underlying the plasma membrane was also detected in BTVΔ_PPRY_ infected cells. This suggested that the abrogation of PPRY domain in NS3 had an effect on the distribution of virus particles. Although our earlier *in vitro* study has demonstrated that the PPRY motif interacts with members of NEDD4 family of E3 ubiquitin ligases [33], this is the first time that the effect of this mutation has been analysed in context of a live BTV infection.

### 3.5. The Conserved PPRY Late Domain in NS3 Influences BTV Growth

The recovered mutant virus was further characterized by infecting PT and control BSR cells with BTVΔ_PPRY_ at low (0.1) and high MOI (1 and 10). Three different MOIs of infection were undertaken to evaluate the effect of increasing MOI on virus growth. The total plaque assay titres were analyzed at 12 and 24 h pi. In addition to WT BTV, a previously described mutant virus (BTVΔ_PSAP_) containing abrogation of the Tsg101 interacting domain in NS3 that affected budding virus release from infected BSR cells was also used to analyze whether it behaved similarly in PT cells [6].

**Figure 6:**
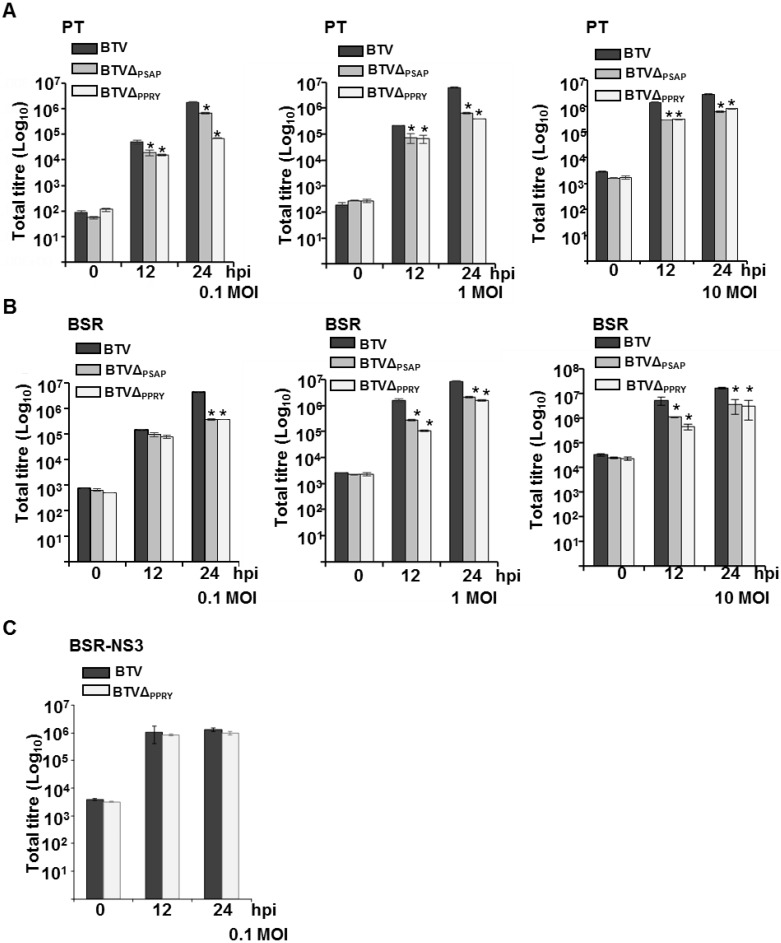
Characterization of BTVΔ_PPRY_ and BTVΔ_PTAP_. Total titres at different times pi of either BTVΔ_PPRY_, BTVΔ_PSAP_ or BTV in PT (**A**); BSR (**B**) and BSR-NS3 (**C**) cells were determined, expressed as PFU/mL, and plotted on logarithmic scales. Asterisk (*) indicates that the decrease in titres of BTVΔ_PPRY_ or BTVΔ_PSAP_ at 12 and 24 h pi is statistically significant to BTV (*p* < 0.05). The MOI of infection, cell types and viruses used have been indicated in each graph.

PT cells infected at low MOI with BTVΔ_PPRY_ showed a significant decrease (asterisk) in total virus titres at both 12 and 24 h pi (*p* < 0.001) (Figure 6A, left). Although the mutant virus BTVΔ_PSAP_ at the same MOI also showed similar effect to BTVΔ_PPRY_ (*p* < 0.001) at 12 h pi, the decrease in BTVΔ_PSAP_ titre at 24 h pi was *p* < 0.05. In comparison, BSR cells infected at low MOI with the two mutant viruses only showed a significant decrease in titre at 24 h pi (*p* < 0.01) (Figure 6B, left). The lack of significant increase of the total titre of BTVΔ_PPRY_ between 12 to 24 h pi in PT cells highlighted the possibility of PPRY domain being more effective at an earlier time point of infection in sheep cells than in BSR. The control mutant virus BTVΔ_PSAP_ has been shown to hamper pinching off the virus particles during budding in BSR cells [6]. Since budding of virus particles has been observed in BSR (Figure 1), but not in PT cells, it is still not clear as to the exact role of Tsg101 in virus release from PT cells. To analyze the effect of MOI on the growth of BTV1Δ_PPRY_, PT and control BSR cells were infected at high MOI of 1 or 10. As previously, BTVΔ_PSAP_ was used as the control. Although the titres of mutant viruses were significantly less than that of WT BTV (*p* < 0.01), there was no significant difference between BTVΔ_PPRY_ and BTVΔ_PSAP_ in both PT and BSR cells (compare Figure 6A, B middle and right). It is possible that the PPRY domain in NS3 is needed for the cell-to-cell spread of the virus particles, a process that is hindered by abrogation of the domain in NS3. When the cells are infected at high MOI, the cell-to-cell spread observed in cells infected with low MOI is most probably overcome by the high titre of the infected virus.

To examine if PPRY mutation in NS3 was directly responsible for this reduction in virus growth, NS3 was provided *in trans* and the complementing cells were infected with WT BTV or BTVΔ_PPRY_ at 0.1 MOI (Figure 6C). There was no significant difference (*p* > 0.05) in the total titres of BTVΔ_PPRY_ and WT BTV, demonstrating that stable cell line constitutively expressing NS3 could compensate for the defect in BTVΔ_PPRY_, and thus substantiate further the role of NS3-NEDD4 family of proteins interactions in virus release.

### 3.6. Mutation of PPRY Domain Alters the Distribution of NS3 in Sheep Cells

To analyze whether the intracellular distribution of mutated NS3 was altered from that of the WT NS3, confocal microscopic analysis of NS3 expression was undertaken in BTVΔ_PPRY_ infected cells immunolabeled with an anti-NS3. Since we intended to determine effect of PPRY domain mutation on early release of virus particles, both PT and BSR (as control) were infected at a low MOI of 0.1.

**Figure 7:**
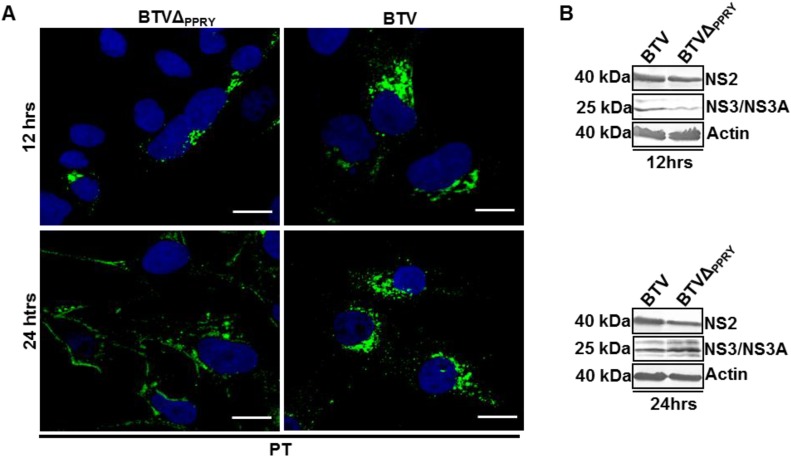

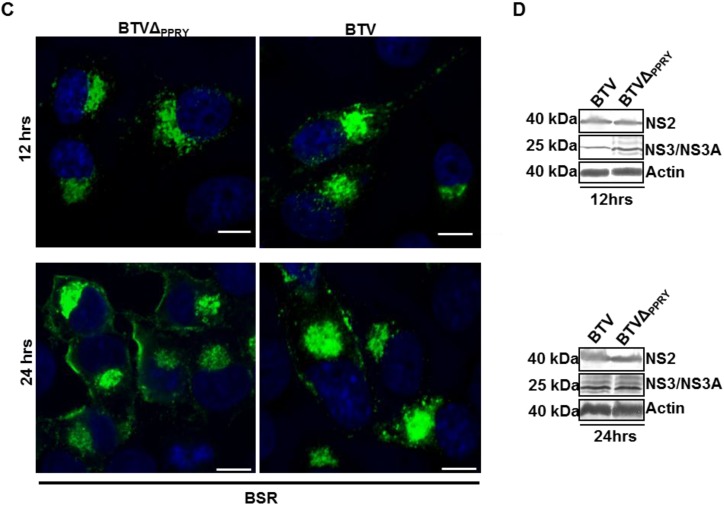
Effect of PPRY domain mutation on NS3 distribution. Localization (**A**,**C**) and expression (**B**,**D**) of NS3 in (**A**,**B**) PT and (**C**,**D**) BSR cells infected with BTVΔ_PPRY_ or WT BTV1. NS3 was visualized in green and nucleus has been labelled with Hoechst (blue). Scale included in each panel represents 20 µm. The expression of NS2, NS3 and actin in infected (**B**) PT and (**D**) BSR cells were analysed at 12 and 24 h pi. Lysates were analyzed by SDS-PAGE and Western blotting. Molecular masses and virus proteins are indicated on left and right, respectively.

Visualization of intracellular distribution of NS3 showed a difference between BTVΔ_PPRY_ and WT BTV infections at 12 and 24 h pi in PT cells (Figure 7A). At 12 h pi, NS3 expression was restricted to juxta-nuclear areas of majority of PT cells (92% ± 1) (Figure 7A, left upper panel). Further, although at 24 h pi, some cytoplasmic punctate expression was observed, strikingly majority of NS3 protein was localized to the cellular margins (85% ± 1.4) (Figure 7A, left lower panel). In comparison, WT BTV infected PT cells analyzed at 12 (82% ± 3.5) and 24 h (83.6 ± 0.7) pi exhibited NS3 in juxtanuclear areas, as small puncta in the cytoplasm and also on the cellular margins (Figure 7A, right). Comparison of BTV protein production at 12 and 24 h pi (Figure 7B), showed that expression of NS3 in PT cells infected with WT BTV was slightly higher than that of BTVΔ_PPRY_ at 12 h pi (Figure 7B, upper panel). However, densitometric evaluation of protein production in infected PT cells did not demonstrate any significant difference (*p* > 0.05) at both 12 (Figure 7B, upper panel) and 24 h pi (Figure 7B, lower panel).

Confocal microscopic analysis of BSR cells at 12 h pi did not show any apparent difference in intracellular distribution of NS3 as observed between BTVΔ_PPRY_ or WT BTV infected cells (Figure 7C). Although at 24 h pi the localization of NS3 to the cellular margins was more prominent for cells infected with the mutated virus than WT BTV, the difference was not as striking as in PT cells (compare Figure 7A,C). Subsequently, when BSR cells were monitored for the expression of NS3, no difference in expression (Figure 7D) was observed in cells infected with WT BTV or BTVΔ_PPRY_ at 12 h pi (Figure 7D, upper panel) and 24 h pi (Figure 7D, lower panel). In all blots the levels of actin, the loading control, were equivalent. Analysis of NS2 expression showed no significant difference in either cell lines (Figure 7B,D). This data confirmed that mutation of PPRY domain did influence the distribution of NS3 protein in PT cells which in turn might affect the trafficking of BTVΔ_PPRY_ particles.

### 3.7. Effect of Mutations in the PPRY Domain of NS3 on Virus Release

To confirm whether mutation of PPRY late domain in NS3 disrupted differential virus egress, the release of BTVΔ_PPRY_ and WT BTV was monitored at 12 and 24 h pi in both PT and BSR cells infected at low (0.1) MOI. To this end, both supernatant and cell fractions were separately harvested and the virus titre of each fraction was determined.

**Figure 8:**
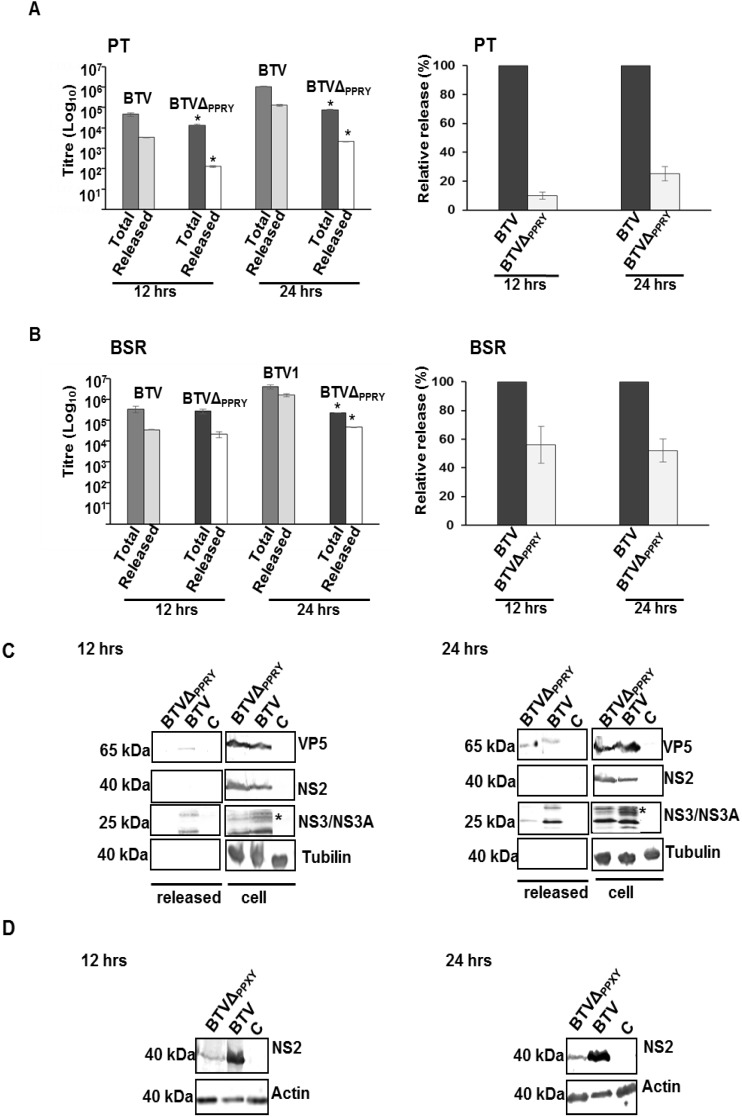
Release of BTV influenced by mutation of PPRY domain. The total and cell free titres in PT (**A**, left) and BSRs (**B**, left) were plotted on a logarithmic scale. The titre of each fraction was determined by plaque assay. (**A**,**B**, right) The relative release was calculated as the ratio of cell free to total virus titre and normalized to 100% for control virus release. Bars represent the standard errors from three sets of replicates; (**C**) Release of virus particles from PT cells infected with BTVΔ_PPRY_ or BTV. Monolayers of PT cells were infected with mutant or control virus. The supernatant and whole cell lysates were harvested 12 h pi (left) and 24 h pi (right) and analyzed by immunoblotting with antibodies against VP5, NS2, NS3/NS3A and tubulin. The protein bands belong to different regions of the same blot. The glycosylation pattern is indicated by asterisk; (**D**) Analysis of NS2 and actin in BSR cells infected with supernatants of PT cells infected with mutant and control viruses, harvested at 12 and 24 h pi.

Although in PT cells, at both 12 and 24 h pi, the total and released virus titres were significantly less than that of WT BTV (Figure 8A, left panel), the decrease was more significant at 24 (*p* < 0.01 and 0.001 for total and cell free, respectively) than 12 h (*p* < 0.05 and 0.01 for total and cell free, respectively) pi. In comparison BSR infected with BTV1Δ_PPRY_ showed significantly less titre at 24 (*p* < 0.01) but not 12 h pi (*p* > 0.05) (Figure 8B, left panel). The ratio of the cell-free to total virus titre also revealed a significant reduction in the relative release for BTVΔ_PPRY_ virus and this effect was more pronounced in PT than BSR cells (Figure 8A,B, right panel). On average, while in PT cells BTVΔ_PPRY_ virus exhibited a decrease of about 10 and 5 fold to WT BTV at 12 and 24 h respectively (Figure 8A, right panel), the decrease in BSR cells at the same times pi was on an average less than 2 fold (Figure 8B, right panel). These results confirmed that abrogation of PPRY motif in NS3 interferes in virus release and that this effect was more pronounced in PT cells.

The composition of the released virus particles was analyzed in BTVΔ_PPRY_ or WT BTV infected PT cells (both at 0.1 MOI). The sum total of BTV particles in the growth media harvested at 12 and 24 h pi were concentrated through a sucrose cushion and analyzed for protein composition. In parallel, whole-cell lysates were evaluated to monitor expression of BTV proteins. Immuno-detection of VP5, an outer capsid BTV protein was used as mature virus particle marker. The non-structural proteins (NS2 and NS3) and tubulin were used to negate contamination of the released BTV particles with cell-associated virus and cellular proteins.

The outer capsid protein VP5 was only detected in the released fraction of BTVΔ_PPRY_ infected cells at 24 h pi (Figure 8C). In comparison, the released fraction of WT BTV1 infected cells contained VP5 at 12 and 24 h pi (Figure 8C). Densitometric analysis of the released proteins at 24 h pi, demonstrating significantly less (*p* < 0.05) expression of VP5 (~60% ± 2%) in BTVΔ_PPRY_ infected cells than WT BTV1 (results not shown), suggested less particle release for mutant virus. As expected, there was no significant difference (*p >* 0.05) of NS2 expression in the cell associated WT BTV and BTVΔ_PPRY_ infected cell fractions (Figure 8C). Interestingly, NS3/NS3A was also detected in the released fractions of BTVΔ_PPRY_ infected cells at only 24 h (Figure 8C), while WT BTV infected cells showed released NS3/NS3A at both 12 and 24 h pi. In addition, the profile of released NS3 was different between BTVΔ_PPRY_ and WT BTV infected cells. The typical glycosylation pattern as seen previously [29] was clearly detectable in NS3 (indicated by asterisk) released from WT BTV infected cells, but not in mutant virus infected cells. It is noteworthy, previous studies have reported association of NS3/NS3A with detergent sensitive structures (membranes) in the growth media of infected cells [28] and with released virus particles surrounded by “transient” envelopes [6].

To prove that the released fractions contained particles and not individual proteins, the concentrated released fractions from infected PT cells were infected in BSR cells and the expression of NS2 was analyzed (Figure 8D). Compared to WT BTV, significantly less (*p* < 0.05) expression of NS2 was observed for BTVΔ_PPRY_ at both 12 (~72% ± 1.5%) and 24 (~60% ± 2%) h pi. The combination of the BTV release assay and the decrease in virus titre demonstrated that abrogation of the PPRY late domain motif in NS3 does influence release of BTV particles.

## 4. Discussion

To date, most studies on virus and host cell interactions have been undertaken in cells that have originated from species that are not the natural virus host. Hence, the exact nature of cellular response to BTV infection in the context of its natural host cell is not well-defined. This study describes BTV infection of PT, a sheep kidney cell line and also elucidates the role of cellular proteins in differential virus release.

Cell sections of infected cells demonstrating a striking association of fully formed virus particles with intracellular vesicular structures in PT cells highlighted the importance of vesicles and membranes in cells that have originated from sheep, the natural host of BTV. Vesicular distribution of enveloped (HIV) and non-enveloped virus particles have been linked to virus assembly, maturation and release [1,52]. Interestingly, while HIV is known to target to MVBs [53], research on picornaviruses have revealed the vesicles as autophagosomes [1,52]. Non-enveloped rotavirus, a member of the *Reoviridae* family, also hijacks the autophagic pathway to traffic viral proteins for assembly of infectious virus particles [54,55].

BTV is known to exit infected mammalian cells by lysis. However, the release of BTV in non-ovine mammalian cells prior to the induction of lysis has been attributed to budding, a phenotype commonly observed in enveloped viruses. The budding phenotype in HIV and other enveloped viruses have been credited to late domains belonging to the ESCRT family of proteins [56,57,58]. Previous studies in BTV have highlighted the importance of Tsg101 interacting late domain in NS3, the only membranous non-structural protein of BTV in BSR cells [6,33]. While no budding was observed in PT cells, the vesicular distribution of BTV in close proximity to the plasma membrane indicated the possibility of vesicles being used as carriers for the trafficking of particles to the cell membrane. In addition, the presence of viral particles within the vesicles suggested the role of these vesicular structures as hubs for the maturation of complete virus particles. Although similar vesicular distribution of BTV have been observed in infected insect vector cells [30], unlike infected mammalian cells, the release of BTV from insect cells do not take place through lysis.

Reports have demonstrated that cellular intraluminal vesicles (ILVs) generated by inward budding of endosomal MVBs [59] can either be targeted for degradation through lysosomal pathways, or MVB may traffic to the plasma membrane, where the ILVs are released into the extracellular space by fusion of the MVB membrane with the plasma membrane. Although it is not clear as to what determines the specific fate of ILVs, it has been well established that the ESCRT machinery is important for the sorting of ubiquitinated cargo into ILVs and for ILV formation [60,61]. Such exosomal vesicles are enriched in cholesterol and PtdIns(3)P group of lipids and raft resident proteins, caveolins and flotillins [62,63]. Exosomes plays an important role in many viruses including rotavirus [64], Hepatitis C [65], Vaccinia [66] and Cytomegalovirus [67]. Our previous study in non–ovine BSR and HeLa cells have also shown that BTV structural and non-structural protein NS3 co-fractionates with caveolins and flotilins which is disrupted by sequestration of cellular cholesterol [40]. Since cholesterol sequestration also decreased the total BTV titre, it was hypothesized that the membrane lipid domains might be important for the final stage of virus maturation, *i.e.*, attachment of the two outer capsid proteins to the core particles [40]. As NS3 interacts with the two outer capsid proteins of BTV, disruption of domains in NS3 affecting its association with membranes will also influence virus assembly. The enrichment of PtdIns(3)P in endosomal membranes and its ability to recruit components of the MVB sorting machinery [68,69] makes it essential for MVB formation [21,70]. Thus, decrease in the relative BTV titre observed on treating infected cells with PI2K inhibitor LY294002 indicated the importance of MVB integrity on BTV assembly.

As ubiquitin is known to mediate vesiculation and virus budding [71,72,73], we treated PT and BSR cells with two proteasome inhibitors, MG132 or lactacystein. Along with degradation of cytosolic and nuclear proteins the ubiquitin/proteasome pathway is also involved in the sorting of various membrane anchored-proteins [74,75,76,77]. In addition, it has been established that proteasome mediated modulation of the levels of free cellular ubiquitin plays an important role in this process [46]. While lactacystin, a *Streptomyces*-derived metabolite is a highly specific inhibitor of proteasome, the tripeptide aldehyde MG132 is less specific. Since we wanted to assess the effect of the inhibitors on virus maturation and early virus release, the cells were infected at a low MOI of 0.1. Growth of BTV in the presence of either of the inhibitors revealed a drastic decrease in relative virus titres and protein production in PT, which was accompanied by a significant reduction in free ubiquitin. Similar results have been obtained for rotavirus where inhibition of proteasome resulted in a detrimental effect on rotavirus titre and protein production, mediated by disruption of rotavirus inclusion bodies formation [78]. Alternatively, as it has been reported that proteasome-ubiquitin pathway influences Rotaviruses replication, the decreased virus titre and protein production in BTV infection can also be due to similar inhibition of BTV replication [50]. However, further investigation is needed to understand the influence of proteasome-ubiquitin pathway on BTV replication. In comparison, when BTV was grown in infected cells for 10 or 14 h and then treated for 2 h with each proteasome inhibitors, although compared to the untreated cells there was a decrease in total titres, there was no effect on virus protein production. This indicated that the short exposure of the infected cells with the proteasome inhibitors either disrupted the maturation of the virus particles or disrupted the release of fully formed mature particles. As before, a reduction in free ubiquitin was also observed. Our studies did not show a significant difference in the ubiquitination pattern of infected cells in the presence or absence of inhibitors. This might be due to the action of ubiquitin not linked directly to ubiquitination of virus proteins, but to some other function, such as regulation of the activity and stability of components of the class E vacuolar protein sorting (VPS) machinery [79]. Studies in enveloped viruses have suggested that although ubiquitin mediates budding of retroviruses and paramyxoviruses, virus and cellular protein ubiquitination is not universally required for their late domain function [79,80,81]. Further experiments on the interplay between ubiquitin machinery and BTV needs to be undertaken to gain an in depth understanding of the influence of the ubiquitin-proteasome pathway on BTV infection.

Along with PSAP, the Tsg101 interacting late domain, NS3 also contains a second completely conserved late domain, PPRY, which binds to NEDD4 family of HECT ubiquitin E3 ligases [33]. Since, HECT ubiquitin ligases functions adaptor proteins that physically bridge PPXY motifs and the class E VPS machinery that tracks protein to the MVBs [79], the effect of the PPXY on BTV life cycle was analyzed. Interestingly, studies with Gag protein of HTLV-1 have reported that Nedd4.1 and Tsg101 act successively to traffic Gag through the endocytic pathway to the late endosomes for retroviral release [82]. While our previous study involving pull down experiments reported interactions of BTV10 NS3 with Tsg101 and NEDD4 family of HECT domain-containing ubiquitin ligases (NEDD4.1, WWP1, and Itch) [33], the late domain function of PSAP was more efficient in facilitating budding of heterologous virus-like particles in 293T cells [33]. Since the effect of the NS3 PPRY domain virus release was not studied in the context of BTV infection, the well-established BTV RG system was utilized to recover mutant BTV that contained mutations abrogating the PPRY domain. Our data showed that the recovered BTVΔ_PPRY_ virus did affect distribution of virus particles and decreased the total and virus release titres. In addition, the alteration of NS3 distribution was more in BTVΔ_PPRY_ infected PT cells. The NEDD4 family of HECT ubiquitin E3 ligase are highly conserved in eukaryotes. Hence, the stronger effect of mutation at PPRY domain mutation in PT cells might be due to the presence or absence of a cellular partner in PT cells that might be having an antagonistic influence in BSR cells. Intriguingly, yeast protein Sna3 utilizes both ubiquitin dependent and independent pathways to transport to the MVBs. Sna3 composed of two transmembrane domains and cytoplasmic amino and carboxy termini is similar in structure to BTV NS3. The late domain PPAY motif in Sna3 interacts with the HECT-type Ubiquitin ligase Rsp5 [81,83] for trafficking to MVB, even in the absence of Sna3 ubiquitination. It has been reported that the ability of Rsp5 to ubiquitinate a number of MVB cargoes and its interaction with ubiquitin sorting machinery might aid in its function as a sorting signal for Sna3 [23]. Since, Nedd4 family of proteins is a human homologue of Rsp5, it is possible that similar actions might be occurring in mammalian cells. However, more studies needs to be undertaken to study this phenomenon in detail.

The importance of PPRY late domain was also confirmed by the difference in the composition of the released BTV particles. This assay showed the presence of membrane protein NS3 in the release fraction. A similar assay undertaken previously has demonstrated association of released NS3 with detergent sensitive entities that were proposed to be cell membranes [28]. In addition, immunogold labelling has also demonstrated association of NS3 with released virus particles that are coated with envelopes [6].

On the basis of our results it can be hypothesized that the influence of cellular factors on early non-lytic release of BTV might be linked to its spread to the internal organs of sheep. This might give the virus more chance to replicate efficiently without causing harm to the infected cells. Ultimately, due to high viral load the cells are lysed which results in the severe damage to the tissues. Whether other non-structural proteins such as NS1 encoded by BTV in infected cells have any role in the vesicular distribution of particles and NS3, remains to be seen. The NEDD4 family of HECT ubiquitin E3 ligase modulated trafficking of BTV particles is different to the function of this late domain in enveloped viruses where it is mainly associated with virus budding [56,58,84,85,86]. While the hijacking of members of the ESCRT family of proteins by BTV non-structural protein NS3 opens up interesting questions on the similarity between non-enveloped and enveloped viruses, this avenue of study is beyond the scope of this manuscript. Further, whether the early non-lytic release of BTV particles mediated by PPRY is linked to cell-to-cell transmission of the virus particles should be investigated in future studies.
